# Image-Guided Laparoscopic Surgical Tool (IGLaST) Based on the Optical Frequency Domain Imaging (OFDI) to Prevent Bleeding

**DOI:** 10.3390/s17040919

**Published:** 2017-04-21

**Authors:** Byung Jun Park, Seung Rag Lee, Hyun Jin Bang, Byung Yeon Kim, Jeong Hun Park, Dong Guk Kim, Sung Soo Park, Young Jae Won

**Affiliations:** 1 Medical Device Development Center, Osong Medical Innovation Foundation, Cheongju, Chungbuk 361-951, Korea; yachon.park@gmail.com (B.J.P.); naviman78@gmail.com (S.R.L.); crisenc@kbiohealth.kr (H.J.B.); nick.kimby@gmail.com (B.Y.K.); pjh8311@kbiohealth.kr (J.H.P.); dgkim@kbiohealth.kr (D.G.K.); 2 Department of Surgery, Korea University College of Medicine, Seoul 02841, Korea

**Keywords:** laser and laser optics, optical frequency domain imaging, optical coherence tomography, laparoscopic surgical tool, medical optics instrumentation

## Abstract

We present an image-guided laparoscopic surgical tool (IGLaST) to prevent bleeding. By applying optical frequency domain imaging (OFDI) to a specially designed laparoscopic surgical tool, the inside of fatty tissue can be observed before a resection, and the presence and size of blood vessels can be recognized. The optical sensing module on the IGLaST head has a diameter of less than 390 µm and is moved back and forth by a linear servo actuator in the IGLaST body. We proved the feasibility of IGLaST by in vivo imaging inside the fatty tissue of a porcine model. A blood vessel with a diameter of about 2.2 mm was clearly observed. Our proposed scheme can contribute to safe surgery without bleeding by monitoring vessels inside the tissue and can be further expanded to detect invisible nerves of the laparoscopic thyroid during prostate gland surgery.

## 1. Introduction

Laparoscopic surgery, which is also called minimally invasive surgery (MIS) with laparoscopy, has become widely accepted as a part of general, gynecological, urological, and thoracic surgeries. Because laparoscopic surgery is performed with a laparoscope and thin rod-shaped surgical instruments through trocars settled on the body wall (the hole size is usually 0.5–1.5 cm), it provides many advantages to the patient compared with open surgery in terms of pain, incision size, and postoperative recovery [1].

While laparoscopic surgery is clearly advantageous in terms of patient outcomes, there are some drawbacks on the surgeon’s side, such as a loss of dexterity, poor depth perception, the fulcrum effect, and a decreased sense of touch [2,3]. In particular, the loss of tactile sensation by depending on tools makes it difficult to avoid invisible blood vessels surrounded by the fatty tissues of the dissection area before resection. In open surgery, surgeons are not only able to feel pulsating blood vessels with their own hands but also use their comprehensive knowledge of gross human anatomy to trace specific local areas that need to be dissected. However, MIS takes away the surgeon’s tactile senses of the tissue and applications of a wide anatomical map due to the magnified local camera view of the laparoscopic system. These inevitable drawbacks of MIS force surgeons to dissect tissues very meticulously to find vessels and expose them until they are nearly naked because they need to confirm them on the monitor and have no tactile sense. Even minor bleeding can make the laparoscopic surgical field very dirty and confusing. This situation sometimes lengthens the duration of MIS. To ensure perfect bleeding control from vessels, surgeons use more hemoclips than they actually need. Moreover, if a blood vessel is not fully captured within the sealing area of the advanced energy device, bleeding cannot be avoided.

The optical frequency domain imaging (OFDI) technique, which is also known as swept source optical coherence tomography (SS-OCT), is a high-sensitivity and high-resolution optical cross-sectional imaging technique based on optical frequency-domain interferometry with a wavelength sweeping laser [4]. Because the optical cross-sectional imaging technique is fast and minimally invasive compared to non-optical techniques such as MRI, CT, and X-rays, it has been extensively studied in a number of medical fields. Clinically, OFDI is used in ocular and cardiovascular applications and has been demonstrated to accurately image the normal eye and coronary artery in vivo as well as diseased states [5,6,7,8,9]. Typically, the penetration depth is about 1–3 mm in tissue, and the depth resolution is about 10–15 µm [10]. OFDI has also been applied to studying the structural features of skin, gynecological tissues, and gastrointestinal tract [11,12,13,14]. Most previous studies on medical devices using OFDI focused on discriminating between normal and diseased or cancerous tissues based on microstructural features. When the OFDI technique is applied in a laparoscopic surgical device such as a tissue dissector, safe surgery without unwanted bleeding can be realized by monitoring blood vessels inside a tissue before resection.

We developed an image-guided laparoscopic surgical tool (IGLaST) to observe blood vessels inside a tissue that uses a clinically qualified OFDI technique and the specially designed laparoscopic surgical tool. We demonstrated our proposed scheme in vivo by observing the blood vessels surrounded by the fatty tissues of a porcine model.

## 2. Materials and Methods

### 2.1. Design of IGLaST

**Figure 1:**
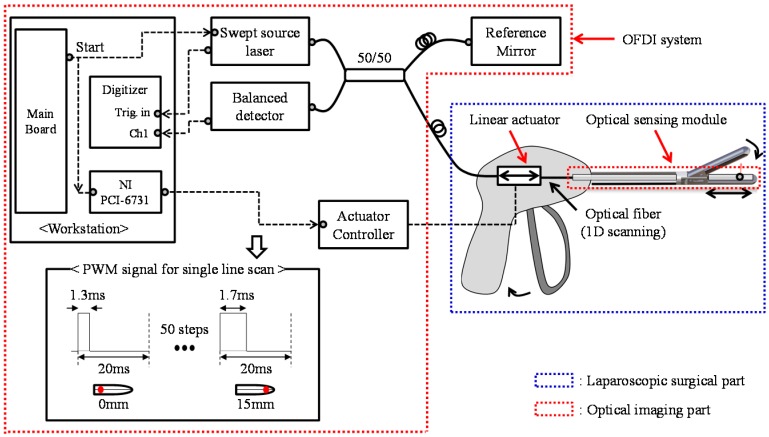
Schematic diagram of an image-guided laparoscopic surgical tool (IGLaST) based on the optical frequency domain imaging (OFDI) technique.

Compared to previous medical devices using OFDI, IGLaST is for laparoscopic surgical devices that grasp a tissue or blood vessel for dissection or sealing. The proposed IGLaST comprises an optical imaging part based on the OFDI technique and a specially designed laparoscopic surgical part, as shown in Figure 1. We designed the laparoscopic surgical part to scan an optical sensing module with a linear actuator and operate the head with a handle. The optical imaging part consists of the OFDI system and an optical sensing module.

Because laparoscopic surgery is minimally invasive, the head size for the surgical instruments is restricted to a diameter of 5–10 mm. The optical sensing module can be simply realized by an optical fiber with a diameter of less than 0.35 mm. Such an optical fiber is inexpensive and easily separated and combined by an optical connector. Thus, OFDI can be an effective technique for realizing an image-guided disposable laparoscopic surgical tool.

### 2.2. OFDI for IGLaST

A swept sourced laser with a center wavelength λ_0_ of 1300 nm and a spectral bandwidth λ_full_ of 100 nm at a cutoff point of −20 dB (SL1310V1-10048, Thorlabs, Sterling, VA, USA) was used as the light source. The swept source is separated by a 3 dB fiber coupler. One of two beams after the 3 dB fiber coupler goes to a reference mirror, and the other goes to the sample. The two reflected beams from the reference mirror and sample pass through the same root in opposite directions and are interfered with. The interference optical signal is detected by a balanced detector and acquired by a digitizer with a sampling rate of up to 500 MS/s at a 12 bit resolution.

In an experiment, the mechanical movement of the optical sensing module was performed by using a linear servo actuator (PLS-5030, POTENIT, Seoul, Korea) for 15 mm transverse line scanning within 1 s. Generally, OFDI imaging requires a high-speed line scanning system (HSLS) for biomedical applications [5,6,7,8,9]. However, HSLS-based OFDI is not required for laparoscopic surgical applications because the sample is tightly fixed by the IGLaST head. Our proposed line scanning scheme with a linear servo actuator provides a low-cost and miniaturized device for practical use in laparoscopic surgical applications. To control the linear servo actuator, an NI PCI-6731 board (National Instruments, Austin, TX, USA) was equipped with a workstation, and pulse-width modulation (PWM) signals were generated as described in Figure 1.

To facilitate clear imaging, the swept source laser, digitizer, and linear servo actuator were synchronized, as represented in Figure 1.

The repetition rate of the light source was 100 kHz, and 100,000 interference signals were generated in a single scan. For a higher signal-to-noise ratio (SNR), the 20 adjacent interference signals were averaged after fast Fourier transform (FFT). Finally, a 5000 × 701 pixel OFDI image was acquired.

### 2.3. Realization of IGLaST

**Figure 2:**
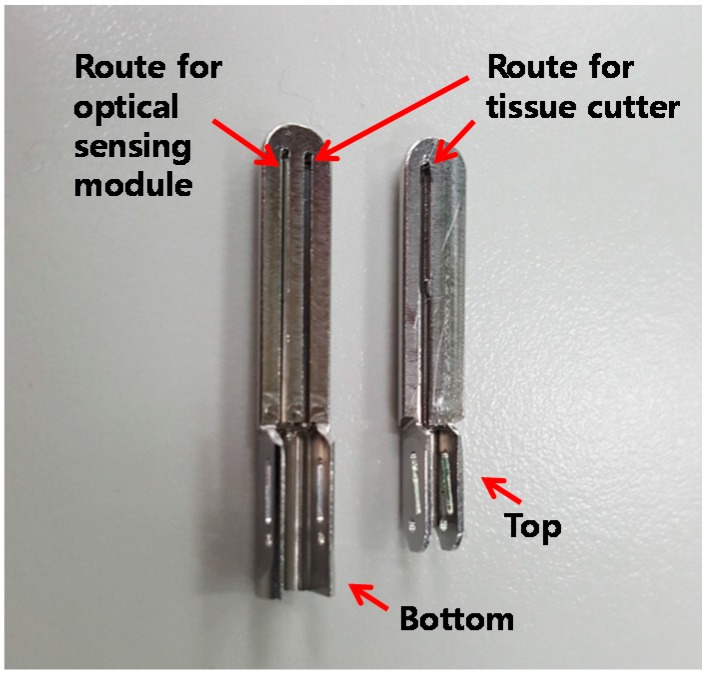
IGLaST head with the route for the optical sensing module.

To apply the proposed method to a laparoscopic tissue dissector, we developed the IGLaST head with an optical sensing module and tissue cutter. The IGLaST head with biocompatible material (SUS304) contains two routes for the optical sensing module and tissue cutter, as shown in Figure 2. The size of the area for grasping the tissue is about 5.3 mm × 20 mm. Each route for the optical sensing module and tissue cutter has the same width of 0.5 mm, and the route for the optical sensing module has a length of 17 mm.

**Figure 3:**
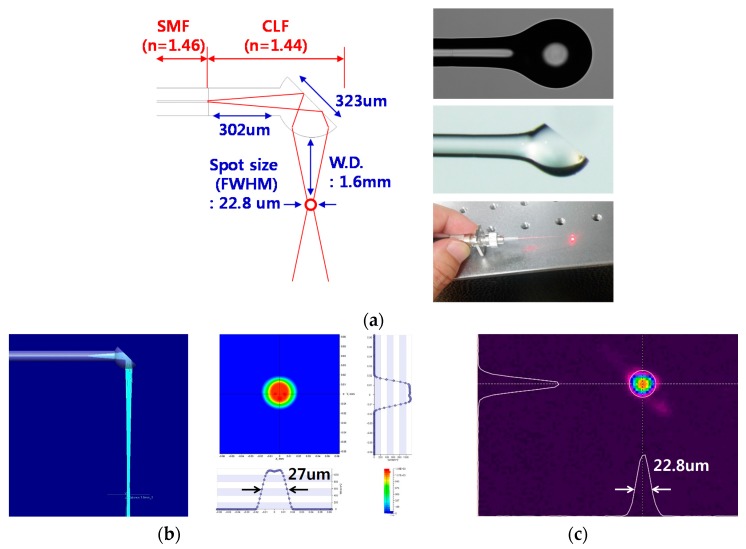
(**a**) Design and manufacture of the ball-lens fiber. (**b**) Simulation results for the ball-lens fiber. (**c**) Experimentally measured beam profile of the ball-lens fiber at a working distance of 1.6 mm.

In order to transmit the laser beam into a sample, a ball-lens fiber functioning as a reflective mirror at the end of a fiber was manufactured, as shown in Figure 3a. This was predetermined by using a simulation tool (Light Tools, Synopsys, Mountain View, CA, USA) to satisfy a spot size (Full width at half maximum) of 27 µm at a working distance of 1.6 mm, as shown in Figure 3b. A coreless fiber (FG125LA, Thorlabs, Sterling, VA, USA) was fusion-spliced to the SMF (SMF-28, Corning, Corning, NY, USA) and cleaved to a predetermined length. Then, the distal end of the coreless fiber was heated, while being translated in a tungsten filament furnace to form a ball lens. The translation length and speed, the temperature of the filament, and the duration of heating were empirically adjusted to form the optimal ball-lens fiber. The entire ball lens fabrication process was performed at a computer-controlled fusion splicing workstation (GPX-3000, Vytran, Morganville, NJ, USA). To perpendicularly reflect the beam into the tissue, the distal tip of the ball lens was polished with a fiber polishing machine (Ultrapol, Ultra Tech., Santa Ana, CA, USA). The angle between the fiber axis and polished surface was about 39°. We confirmed the spot size (FWHM) of the beam was about 22.8 µm at a working distance of 1.6 mm by using a beam profiler (SP620U, Spiricon, Jerusalem, Israel), as shown in Figure 3c. The distance between the SMF/coreless fiber interface and front of the ball lens was 302 µm, and the coronal diameter of the ball lens was 323 µm.

**Figure 4:**
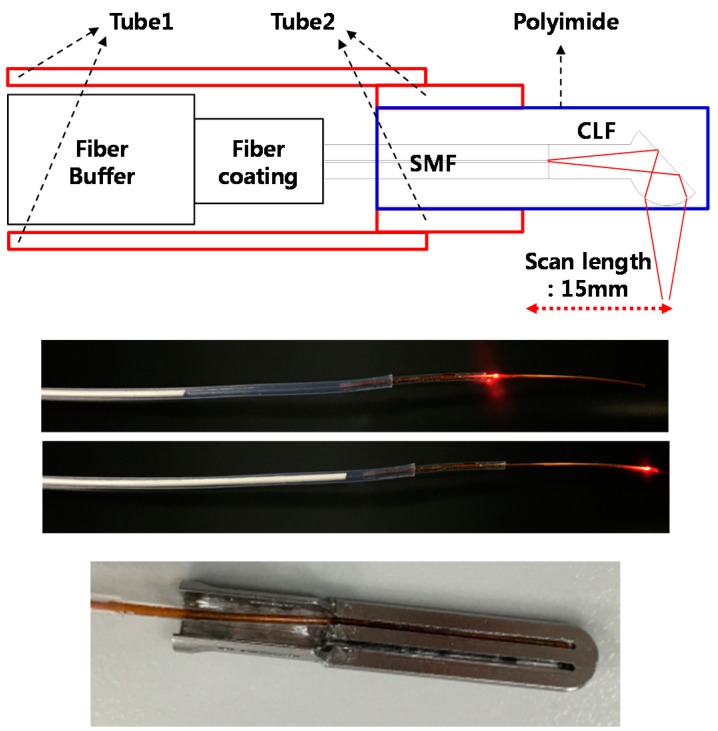
Optical sensing module for IGLaST.

Figure 4 shows the structure of proposed optical sensing module. To protect the ball-lens fiber, a polyimide with an inner diameter of 350 µm and outer diameter of 390 µm was used. The polyimide was inserted into the groove on the bottom of the IGLaST head and secured with epoxy, as shown at the bottom of Figure 4. The rest of the ball-lens fiber was guided by Shrinkable Tube 1. Between the polyimide and Tube 1, Shrinkable Tube 2 was used as a spacer and had a smaller inner diameter than Tube 1. The length of Tube 1 was about 500 mm, which is similar to the length of a laparoscopic surgical tool. After the end of Tube 1, a linear servomotor was used for one-dimensional scanning of the ball-lens fiber. To minimize twisting or bending of the ball-lens fiber, the linear servomotor was placed close to the end of Tube 1.

One-dimensional scanning of the ball-lens fiber inside the optical sensing module was tested by using a laser diode with a central wavelength of 633 nm, as shown at the middle of Figure 4. After the optical sensing module was assembled, the head was attached to the IGLaST body.

## 3. Results

### 3.1. Optical Properties

**Figure 5:**
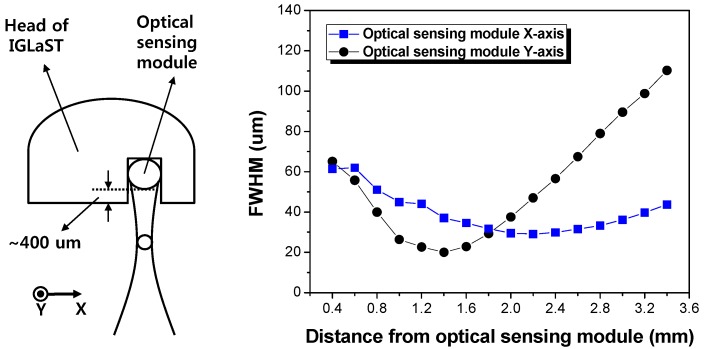
Beam diameter (FWHM) of the optical sensing module.

Figure 5 plots the experimentally measured beam diameter (FWHM) of the optical sensing module of IGLaST. The beam shape was measured by using a microscope equipped with an IR camera [15]. The beam diameter plot started at 400 µm considering the air gap between the optical sensing module and IGLaST head surface. The beam profile was elliptically shaped after passing through the polyimide. The ratio of the *x*-axis/*y*-axis crossed at the focal plane. The measured *x*- and *y*-axis beam diameters were 29.3 and 31.8 µm, respectively. The lateral resolution expected by the FWHM beam width ranged from 20 to 65 µm in the first 2 mm after the IGLaST head.

### 3.2. Imaging of a Vessel Inside the Fatty Tissues of a Porcine Model with IGLaST

As a demonstration, IGLaST was used to observe blood vessel inside fatty tissue during laparoscopic surgery. We prepared a 40 kg porcine model that was 3 months old. The pig underwent surgical procedures under general anesthesia. Three incisions with a length of 0.5–1 cm were made on the abdomen of the pig, and trocars were placed in the incisions. A laparoscope, laparoscopic dissection tool, and laparoscopic clamp were inserted inside the body through the trocars, and an operation was performed to find the region of interest. The animal experiment protocol was approved by the Institutional Animal Care and Use Committee (IACUC) of Korea University.

**Figure 6:**
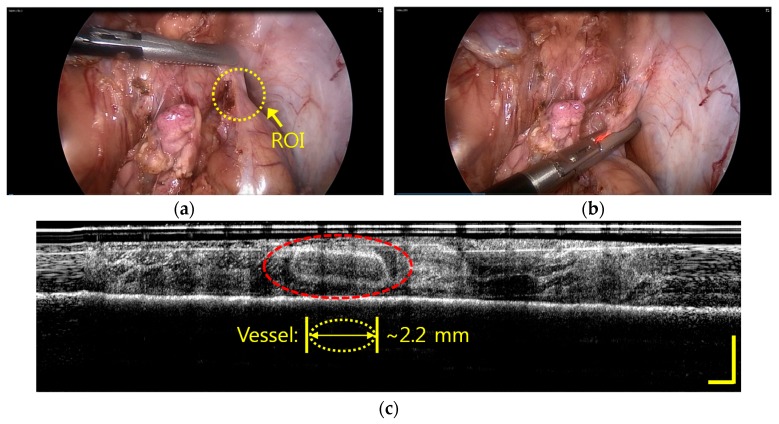
In vivo imaging inside the fatty tissue of a porcine model with IGLaST; the blood vessel inside the tissue is visible. (**a**) Laparoscopic image of the porcine model. (**b**) Laparoscopic image of the porcine model after the tissue is grasped with IGLaST. (**c**) OFDI image inside the fatty tissue.

Figure 6 shows the in vivo imaging inside the fatty tissue of the pig with IGLaST. In laparoscopic surgery, tissues to be dissected should consist of adipose, lymphatics, and collagen, and blood vessels must be surrounded by the tissue complex. Therefore, the tissue complex that may cover major blood vessels of an artery or vein usually needs to be dissected to find them. In most cases, blood vessels inside the tissue are invisible, as shown in Figure 6a. We inserted our developed IGLaST inside the pig body through a trocar and grasped the tissue with the head of IGLaST to observe invisible blood vessels inside the tissue, as shown in Figure 6b. The red light is a guide source to inform the position of the head of the ball-lens fiber. The blood vessel inside the fatty tissue of the pig appeared with OFDI, as shown in Figure 6c. The yellow bar in Figure 6c represents a length of 0.5 mm. The size of the image was about 15 × 1.75 mm^2^. After the tissue was grasped, the tissue was compressed, and the thickness of the tissue was reduced to about 0.5–0.6 mm. In this experiment, a blood vessel with a length of about 2.2 mm was clearly observed.

**Figure 7:**
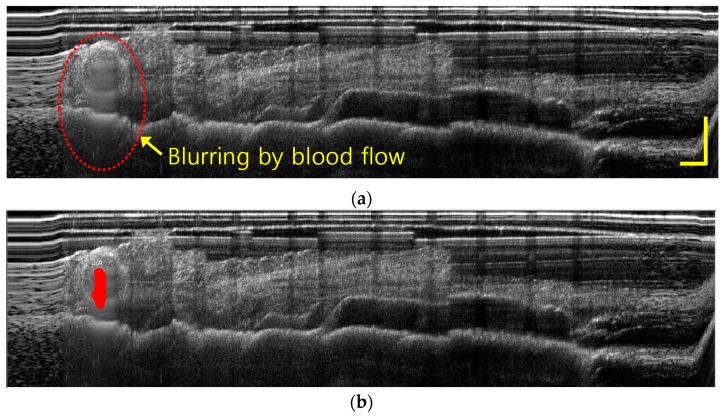
(**a**) In vivo imaging of the blood flow inside the fatty tissue of a porcine model with IGLaST. (**b**) Identification of blood flow with the morphological image processing technique.

When the IGLaST head grasping the tissue was slightly released, image blurring occurred at the vessel position because of blood flow, as shown in Figure 7a. If the morphological image processing technique to extract blurring pixels is applied, the contrast can be increased to improve awareness of the blood vessels inside the tissue, as shown in Figure 7b. This technique provides the functions of erosion, smooth filtering, simple threshold, and area sort to isolate the blurred part [16]. IGLaST can be further improved to discriminate arteries and veins by the application of Doppler and angiographic techniques [17,18,19].

## 4. Discussion and Conclusions

We proposed and developed IGLaST based on the OFDI technique to prevent bleeding. To observe within tissue that may cover invisible blood vessels of a vein or artery, we specially designed a laparoscopic surgical tool and applied the OFDI technique. We successfully demonstrated that our proposed scheme can be used to prevent unwanted bleeding by observing a blood vessel with a diameter of about 2.2 mm inside the fatty tissues of a porcine model during laparoscopic surgery. The experimental results suggest that IGLaST can be a useful tool for investigating blood vessels inside the tissue before resection. The utility of IGLaST may extend to other surgical devices such as laparoscopic surgical staplers, laparoscopic vessel sealing devices, and surgical scissors.

The use of a low-speed linear servo actuator may be unfamiliar for researchers interested in high-speed OFDI-based medical devices for cardiovascular imaging and ocular imaging. However, it is sufficient to prove the feasibility and usability of IGLaST because the sample was tightly fixed by the IGLaST head. The proposed line scanning scheme with a linear servo actuator provides a low-cost and miniaturized device for the practical use of laparoscopic surgical applications. In future IGLaST designs, we will apply the high-speed OFDI technique to the laparoscopic surgical tool to identify the blood flow of blood vessels inside the tissue so that arteries and veins can be discriminated based on Doppler and angiographic techniques.

For the optical sensing module, a beam diameter (FWHM) of less than 65 µm was obtained in the first 2 mm after the IGLaST head. In this study, the size of the observed blood vessel inside the tissue was a few millimeters, so the achieved lateral resolution was acceptable. However, the lateral resolution of IGLaST needs to be further improved to observe small blood vessels or nerves inside the tissue.

We believe that techniques using IGLaST can also be applied to identify invisible nerves or lymphatic vessels inside the tissue to avoid injuring them during minimally invasive surgery (MIS). Moreover, our proposed scheme has tremendous potential as a smart image-guided laparoscopic surgical tool for robot-assisted surgery.
